# The Flora of Chad: a checklist and brief analysis

**DOI:** 10.3897/phytokeys.23.4752

**Published:** 2013-05-13

**Authors:** Giuseppe Brundu, Ignazio Camarda

**Affiliations:** 1 Department of Science for Nature and Environmental Resources (DIPNET), University of Sassari, Via Piandanna 4, 07100 Sassari, Italy

**Keywords:** Chad, Flora, Plant Diversity, Checklist

## Abstract

A checklist of the flora of Chad has been compiled by the authors, based on literature, on-line data-bases, herbarium collections and land surveys (1998-2011). It counts 2,460 records, i.e. 2,288 species (including 128 autonyms), 83 subspecies, 81 varieties, 8 forms, while all the previous available information reported 1,600 species. They belong to 151 Families, with 48.7% of the taxa belonging to the 6 largest families, i.e. *Poaceae* (14.6%), *Fabaceae* (13.6%), *Cyperaceae* (7.0%), *Asteraceae* (6.2 %), *Malvaceae* (3.9%) and Rubiaceae (3.4%).

A total number or 2,173 species (88.3%) are native to Chad, including 55 (2.2%) endemic species, while 274 (11.0%) are alien to Chad, and 13 (0.5%) are considered cryptogenic, i.e. of uncertain status. It represents a considerable update on previous knowledge on the alien flora of Chad that counted for 131 taxa (5.3%). There are 657 therophytes (26.7%), 546 phanerophytes (22.2%), 378 hemicryptophytes (15.4%), 256 chamaephytes (10.4%), 160 geophytes (6.5%), 107 helophytes (4.3%), 104 hydrophytes (4.2%). A total of 252 taxa (10.2) may have different life forms (e.g. terophytes or chamaephytes).

## Introduction

Plant diversity provides numerous essential services to society. These include material goods (for example, food, timber, medicines, and fiber), ecosystem functions (flood control, climate regulation, and nutrient cycling), and nonmaterial benefits such as recreation. Plant diversity contributes to agriculture (wild crops relatives) and forestry, provides carbon storage and sequestration (Millennium Ecosystem Assessment 2005, Power 2010). Plant diversity and biodiversity in general also secure long-term flows of benefits from nature by providing resilience to disturbance and environmental change (Maestre et al. 2012). These and other economic and social contributions are substantial, with recent estimates claiming that the economic value of benefits from biodiverse natural ecosystems may be 10 to 100 times the cost of maintaining them (Rands et al. 2010).

The floristic inventory of a region or country is a very first basis, a necessary prerequisite for assessing plant diversity and for much fundamental research in botany and plant ecology, such as modeling patterns of species diversity or understanding species distribution and highlights key conservation issues.

The studies on the flora and vegetation in Chad started around the ‘50s with the relevant pioneer contributions of R. Corti, H. Gillet, R. Maire and P. Quézel. Although their great efforts and fundamental contributions posed the basis of the knowledge of flora and vegetation in Chad, botanical exploration has been not yet reasonably comprehensive, the more recent (partial) checklists and maps are mainly dated to the ‘70s (see references cited therein) and an updated flora, or even an updated country checklist are lacking. Botanical studies has been interrupted for a long period, also due to the civil war, with the result that the available base of knowledge is quite dated and limited to a few regions of the Country. As a result, Chad is one of the African countries whose flora and vegetation are less studied and known. Chad territory is greatly extended in latitude, several climatic zones and vegetation belts are found, along with very different peculiar habitats and land uses, and therefore significant biodiversity richness is to be reasonably expected.

According to Davis et al. (1986) and Stuart and Adams (1990), as cited in Keith and Plowes (1997), there are about 1,600 species of vascular plants in Chad, of which 1,516 occur south of about 16 N latitude. This is expected to be a very low number considering the land size and the diverse climatic conditions in Chad. The Tibesti Mountains are estimated to have about 450 species of plants, with a mix of Mediterranean, Saharan, Sahelian and afromontane elements (Davis et al. 1986). Lacking additional further studies, these figures (1,600 species) were confirmed later in the comprehensive work of Frodin (2001) on standards Flora of the word and in several reviews on global plant diversity (e.g., Davis et al. 1994–1997, Groombndge and Jenkins 2002, Pitman and Jørgensen 2002).

During the years 1998-2011 the Department of Botany of the University of Sassari, in collaboration with the University of N’Djamena have investigated the flora and vegetation of Chad, with land surveys and literature and herbaria research and analysis (Camarda et al. 2004a, Camarda and Brundu 2005).

The present checklist of the Flora of Chad has been compiled as a first step to making more accessible the data collected and still unpublished, or already published but little known, in order to facilitate subsequent taxonomic work and further botanical research and biodiversity conservation planning. In fact, the literature is much dispersed and is often difficult to access because most of the papers are relatively old and/or were published in regional or national journals which are not readily available.

This paper is not a pure taxonomic work. Not all type materials have been studied and the authors of this paper can not give their opinion about the taxonomic correctness of all the recorded taxa.

## Methods

### Study area

Chad is a medium-size, land-locked African country, with low population (1,284,000 km^2^, more then 7 millions of habitants, density 4.9 habitants/km^2^, with more than 1 million people living in the capital). The Sahelian and Ennedi regions are mainly disabitated (0.1 habitants/km^2^) yet subject to unpredictable and variable pressure of nomads. Most of the population is concentrated in the Southern part of the Country, i.e. around lake Chad basin, Chari and Logone floodplains (54), and in the urban areas of N’Djamena, Bongor, Moundou and Sarh. Chad territory is greatly extended in latitude. Average annual rainfall decreases with increasing latitude, from 1,150 mm at Moundou (08°30'N) to 902 mm at Am Timan (11°N), 582 mm at N’Djamena (12°N), 43 mm at Faya-Largeau (18°N) and no reliable rainfall further north (Scholte and Robertson 2001). It is important to note that a quite large proportion of the country in the north receives less then 100 mm of rainfall per year, and that 4 of the 5 main African climatic zones are present in Chad, notably hyper-arid (< 100 mm), arid (100–200 mm), semi-arid (200–400 mm), sub-humid dry (400–800) and sub-humid humid (800–1,300 mm).

The dry season in the south extends from November to March and increases in duration northwards. Reflecting the decreasing south–north rainfall gradient, the vegetation of the country is divided latitudinally into three sub equal zones and a minor fourth in the southern part: the Saharan desert belt in the north (including the Saharomontane vegetation of Tibesti), the Sahelian belt covering the central third (Semi-desert grassland and thorny shrubland to wooded grassland and bushland, with *Acacia* spp., *Commiphora africana*, *Balanites aegyptiaca*, *Euphorbiaceae*, and abundant dryland taxa), Sudanian belt (woodland and dry forest, with *Celtis integrifolia*, *Hymenocardia acida*, *Lannea* spp., *Prosopis africana*, *Mytragyna inermis*) in the south and a minor area of Sudano-Guinean vegetation, as a mosaic of dry, peripheral, semi-evergreen rainforest and woodland or secondary grassland, regarded as transitional between the Sudanian and Guineo-Congolian vegetation (Pias 1970, Olago 2001). Land uses, land cover (Mayaux et al. 2004) and agro-ecological zones in Chad, as in the most of Western and Central Africa (Jalloh et al. 2012), are closely related to the above cited climatic zones.

The northern third of the country forms part of the Sahara desert and includes, on the border with Libya, the Tibesti massif which rises to 3,415 m. This volcanic massif is the highest both in Chad and the Sahara. Tibesti covers an area of about 50,000 km² and has an average altitude of over 2,000 m. There are areas of Saharomontane vegetation on the massif which are floristically rich and unrelated to the vegetation of the surrounding lowlands (Maire and Monod 1950, Bruneau de Miré and Quézel 1961, Scholte and Robertson 2001) while woody vegetation occurs in some deep gorges. Situated south-east of Tibesti, on the Sudan border, is the Ennedi massif, which rises to 1,450 m with sited of great environmental concern such as Guelta of Archei (Camarda et al. 2004b). The Sahelian belt occupies the middle third of the country. It is bounded in the west by the banks of Lake Chad, the fourth-largest lake in Africa (between 6° to 24°N and 8° to 24°E) and a trans-boundary Ramsar site of international importance. Approximately half of the lake lies within Chadian borders while the rest is shared between Nigeria, Niger and Cameroon. The portion of Lake Chad belonging to Chad is evaluated in 16,481.7 km^2^ (COP8 2002). During the normal period, three types of landscapes are to be seen: the open waters with scarce aquatic vegetation (*Nymphaea* sp.), the reed-islands which are islands of vegetation (e.g. *Cyperus papyrus*, *Phragmites australis*, *Aeschynomene* sp.pl.) and the archipelagoes consisting of about a thousand sandy islands corresponding to the dune crests of a settled, partly immerged Erg (Carmouze et al. 1983, Dupont 1970, Leveque 1983, Olivry et al. 1996). Lake Fitri, the country’s second largest wetland, has been called a miniature lake Chad. This Biosphere Reserve is a shallow lake and completely dried up in 1984-1985 after years of drought (Keith and Plowes 1997). The lake has a productive fishery that is important to the local economy and also a remarkable presence of water birds and interesting vegetation communities, typical of Sahelian wetlands.

On the opposite side of the country, on the Sudan border, and located to the south of the Ennedi massif, the Ouaddai massif rises to a maximum height of 1,260 m. Much of the area between Lake Chad and the Ouaddai massif is, except for parts of the centre-west where there are large expanses of dunes fringed by xerophytic scrubland, a vast, relatively featureless plain (300–400 m) supporting Sahelian grasslands (Scholte and Robertson 2001). Much of this area is drained by the seasonal Batha river which originates from streams running westwards from the Ouaddai massif and which ultimately empties into the temporary wetlands around Lake Fitri (12°50’N, 17°30’E). Indeed, an area of 10 million ha in the transition zone between the southern Sahel and northern Sudan–Guinea is subject to regular seasonal inundation (Scholte and Robertson 2001). Other than the Guéra massif, situated in the centre-north, of which the highest peak reaches 1,613 m, the area is mostly flat, lying mainly between 300 m and 400 m. The main rivers of this zone are the Chari and the Logone, which flow north-westwards and drain into Lake Chad.

The soils in the West-African region have been surveyed for several years by teams of African and European soil scientists. Charreau (1974) published the first most exhaustive soil classification for the Sub-Saharan Africa. The West-African soil map was made of 12 classes subdivided into sub-classes, groups and sub-groups, families, series and types of soil. The family was composed of soils originating from the same kind of parent material. The series related to the position of the soil on the toposequence and the types to the texture of the surface horizon. Sub-Saharan Africa was divided into five large zones (Charreau 1974, Fellera et al. 2008). According to the more recent classification of major soils types of Africa (Panagos et al. 2011) which follows the WRB system (World Reference Base for Soil Resources) the most common soils in Chad are *Leptosols*, *Regosols* and *Arenosols* in the central and northern regions, *Fluvisols*, *Plinthosols*, *Planosols*, *Solonchaks*, *Vertisols* in the southern part.

In Chad 83% of the working population is engaged in the production of crops and livestock, primarily for domestic consumption. Only 2% of the land is cultivated, but about 50% is grazed. Cereal grains are the main food crops, while beans, corn, rice, vegetables, dates are important in local areas. Cotton and peanuts are the primary cash crops (Arditi 1995, Keith and Plowes 1997, Pret 1993, Stuart and Adams 1990). The main sustenance crops are rain-fed, cultivated and harvested by hand, and growth without the use of fertilizers and other agrochemicals which are used on rice and cotton (Keith and Plowes 1997).

According to FAO/UNEP (1981) assessment, the total forest surface in Chad in 1980 was composed by 130,000.0 km^2^ of open forest, 5,000.0 km^2^ of closed forest (in the South), and 30.0 km^2^ of plantations (e.g. *Azadirachta indica*, *Eucalyptus* spp.). These estimates are part of the United Nations Global Environment Monitoring Systems (FAO, 1986). These values are only indicative, because there has not been any National inventory. More recently Tal (2001) reports differing estimated values, *i.e*. 3,626,000 ha of open forest, 211,000 of closed forest, 9,412,000 ha formations with sparse trees, and 10,192,000 of “thickets” or “bushlands”. Plantations are planned to be increased to reach 10,000 ha (FAO, 2001). This last estimates and classification systems seem a little more reliable. The Sahel is characterized by semi-desert grassland, thorn scrub and wooded grassland dominated by *Acacia* sp pl. (Wickens and White 1979, White 1983, Wickens 1984). Trees in and shrubs the Chadian Sahelian belt are important as sources of fuel and timber and are becoming, somewhat belatedly, recognized by foresters and administrators as important sources of such non-wood forest products as food, browse, medicines, fibre, etc. (Wickens 1991). Desertification is widespread throughout the Sahelian zone and there is no quick fix solution to what is an exceedingly complex ecological, geographical, sociological, political and economic problem (Wickens 1997).

The natural forest of *Gam* stretches over more than 5,500 ha and it is indicated as the largest forest in Chad (Pelloux and Boykas 1997). Located in the south, it is threatened by the increasing demand for building materials, as a result of the growing urbanization around the main centers. The *Assale* forest is situated in the Sahelian-Sudanese transition area, around lake Chad, about 110 km North of N’Djamena (Boykas et al. 1997). The growing demand for fuelwood in the capital and the relatively recent arrival of the asphalt road, have aroused concern about the overexploitation of the forest. As in most of Africa, the tree resource base is deteriorating. Population growth is putting heavy pressure not only for fuelwood but also for livestock grazing and crops, and these affects are synergetic with climatic stocasticity. Thus there is a strong need to plan conservation of forest genetic resources both *in situ* and *ex situ* (Tal 1994) and set up management plan and education activities.

### Land surveys

Ten main botanical field surveys and additional short visits and excursions, including boat surveys, have been done by the Authors in Chad in period 1998-2011. These land surveys aimed to explore the main protected areas of the Country, and most of the regions and sites of environmental concern [Borkou-Ennedi-Tibesti (BET), Ounianga Kebir salt lakes, Faya, Fada, Guelta d’Archei, Kanem, Lake Fitri, Batha, Biltine, Ouaddai, Lake Chad, Chari river, Chari-Baguirmi, Guéra, Salamat, Zakouma National Park, Mayo-Kébbi and Logone flood plain], for more than 20,000 km of tracks. The lack of a reliable road network severely limits the possibility to survey the whole Country. Chad is heavily reliant on its 32,000 km road network for the transport of agricultural goods and for linking its widely-dispersed population. However, only around 300-km of the roads are paved, with the rest consisting of poorly-maintained earth or track. Severe flooding during the rainy season renders these roads impassable. During the botanical surveys remarkable sites or species presence and assemblages have been located by hand held GPS, and specimens collected for a dedicated herbarium.

### Literature sources

All the available literature sources documenting presence and distribution of plant species in Chad have been analyzed and data extracted for the compilation of the present check-list (e.g. Audru 1966, Bruneau de Miré and Quézel 1961, Corti 1942, Gaston 1966, 1967, Gillet 1959, 1960, 1961, 1962, 1963, 1968, 1969, Hutchinson and Dalziel 1954-1972, Lebrun and Gaston 1976, 1977, 1986, Lebrun et al. 1972, Maire and Monod 1950, Maley 1981, Mosmier 1963, Pias 1970, Quézel 1957, 1958a, 1958b, Scholz 1966).

As remarked in the introduction section, this paper is not a pure taxonomic work. Not all type materials have been studied and the authors of this paper can not give their opinion about the taxonomic correctness of all the recorded taxa. The main intention has been to bring together the country data in order to facilitate subsequent taxonomic work.

It has been assumed that if one plant is synonymised with another, then its distribution data can also be transferred. When names from the source lists have been synonymised, the original names under which taxa were published are included as synonyms and their distribution data (presence/absence for Chad) are shown under the current name. No distinction has been made between nomenclatural and taxonomic synonyms. Whenever available, specific studies have been taken into account, e.g. Romo and Boratyński (2011) for genus *Luzula* (Juncaceae), Anthelme et al. (2001) for Ferns in Sahara Mountains, Montes-Moreno et al. (2010) and Qaiser and Lack (1986) for the genus *Phagnalon*, Kaplan and Symoens (2005) for *Potamogeton*, Beier (2005) for *Fagonia*.

### Herbarium data and on-line data-bases

Most of the specimens collected and studied by the botanists that performed botanical surveys in the past are actually not in Chad but spread elsewhere, e.g. in Algeria, and have not always been preserved, e.g. in Berlin-Dahlem the collection by Gustav Nachtigal, the first botanist that collected specimens in Chad, was destroyed during the 1943 war, as reported by Lebrun et al. 1972. Important herbaria housing numerous material from Chad are at Dakar (Herbier de l’Institut Français d’Afrique Noire, see Maire and Monod 1950), at Florence (samples collected by Monterin in 1934, were studied by Roberto Corti and stored in the Erbario dell’Università di Firenze, Italy, as reported by Corti 1942), at Maisons-Alfort (Institut d’Elevage et de Medicine Veterinarie des Pays Tropicaux, Maisons-Alfort, France, ALF, see Lebrun et al. 1972), at Paris (Muséum National d’Histoire Naturelle MNHN, Paris, e.g. see Gillet 1968), at Poznan, Poland (collection by Stanislaw Lisowski stored in the Laboratory and herbarium of tropical plants, A. Mickiewicz University, POZG), at Tripoli (Natural History Museum, collection by A. Desio, as reported by Corti 1942), at Kew (collection in the Southern Tibesti by Shaw, as reported by Corti 1942). Additional information on collection in Chad is also reported by Hepper and Neate 1971. Nevertheless, this geographic hindrance to visit different herbaria, is today somewhat reduced thanks to the availability of web pages, such as the one of MNHN of Paris or the ALUKA and JSTOR projects, and thanks to international projects of biodiversity data sharing such as the Global Biodiversity Information Facility (GBIF, http://www.gbif.org/).

All these internet sources have been checked and they have been really useful for the purposes of the present research. For example, the GBIF biodiversity occurrence data published for Chad, was accessed through GBIF Data Portal *data.gbif.org* on the 30^th^ of November 2012 and downloaded. It is not new that internet resources enable and simplify taxonomic work and support filling gaps in biodiversity knowledge (e.g., see Smith and Figueiredo 2010).

The only *herbarium exsiccata* which are presently stored in Chad are those at the University of N’Djamena, collected by S. Lisowski and collaborators, and in Farcha (Laboratoire de Recherches Veterinaires et Zootechniques de Farcha, N’Djamena, Chad) housing about 8,000 samples for a total number of about 1,500 taxa (estimation done by Yosko et al. 2002), collected mainly by A. Gaston and collaborators (Yosko et al. 2002). Although they represent very important documentary resources, these two herbaria store samples coming only from a few regions of Chad and data is not available in digital form. The collections done by A. Melis, I. Camarda and G. Brundu (1998-2011 surveys) are stored at the University of Sassari, Department of Science for Nature and Environmental Resources (SS herbarium).

The data-base of the ALF herbarium is accessible through the dedicated portal. Accessing on the 30^th^ of March 2012 we retrieved a total of 4,191 records, i.e. herbarium samples collected in Chad (http://alf.plantnet-project.org/search) and maintained by CIRAD France (Département d’Élevage et de Médecine Vétérinaire du CIRAD).

The WSCP on-line data-base (World Checklist of Selected Plant Families, facilitated by the Royal Botanic Gardens, Kew, published on the Internet; http://apps.kew.org/wcsp/) was retrieved on the 30th of March 2012, the Kew Grass-Base (Clayton, W.D., Vorontsova, M.S., Harman, K.T. and Williamson, H. - 2006 onwards. GrassBase - The Online World Grass Flora. http://www.kew.org/data/grasses-db.html) was accessed on the 30th of March 2012, the Catalogue of life (that includes records from the following databases *Droseraceae* Database, ELPT, GCC, ILDIS, IOPI-GPC and WCSP) was accessed on the 06th of August 2012, the African Plants Database (version 3.4.0, Conservatoire et Jardin botaniques de la Ville de Genève and South African National Biodiversity Institute, Pretoria, http://www.ville-ge.ch/musinfo/bd/cjb/africa/) was accessed on the 30th of March 2012.

### Nomenclature

The checklist follows the revised and updated classification of the flowering plants at the ranks of orders and families published by the Angiosperm Phylogeny Group (APG III 2009). Families are arranged according to the linear sequence LAPG III by Haston et al. (2009), while the paper by Chase and Reveal (2009) provides a classification scheme with an arrangement of the classes, subclasses, and superorders of extant land plants. The linear sequence of families and genera of ferns follows Christenhusz et al. (2011).

Plant names have been verified using IPNI (International Plant Name Index, http://www.ipni.org/), The Plant List (2010, version 1, published on the Internet; http://www.theplantlist.org/), WCSP and the African Plants Database (APD, version 3.4.0), updated by the Conservatoire et Jardin botaniques de la Ville de Genève and the South African National Biodiversity Institute, Pretoria, South Africa (http://www.ville-ge.ch/musinfo/bd/cjb/africa/).

The main synonyms, the literature source and other information (whenever available) have been stored in the database and are shown in the present checklist as a separate synonyms table. We have given, to our best attempt, the accepted and correct nomenclature according to current taxonomic standards.

### Life forms

In his study on flora and vegetation of the Ennedi, Gillet (1968), using a modified classification system derived from Raunkiaer (1954), describes 7 main life forms for the 526 taxa recorded in the Ennedi (41.3% therophytes, 3.0% geophytes, 5.1 % helophytes, 1.1 % hydrophytes, 7.0% hemicryptophytes, 18.4% chamaephytes, 15.1 % phanerophytes) and 2 additional forms, i.e. parasites (0.9%) and *plurisaisonnières* (8.1%) plants, with the latter defined as annual or pluriannual species. These 9 life forms are divided into sub-forms, for a total of 56 types. There are of course limits to use the Raunkiaer system in tropical and subtropical regions (e.g. see Proches et al. 2005; Harrison et al. 2010). For example, it could be pointed out that some Chadian plants can be classified as belonging to several morphological types, e.g. geophytes and climbers [e.g. *Kedrostis* (Cucurbitaceae), *Dioscorea* (Dioscoreaceae) ], or geophytes and succulents, or to several life histories (Gillet 1968). When recording the life form we took into account the description from Gillet (1968), the paper from Poilecot (1999) for Poaceae and the African Plants Database for all the other taxa.

We have considered the following six categories for life-history: annual (A), perennial (P), biannual (B), annual or biannual (AB), annual or short-lived perennial (AS), and annual or perennial (AP). Life forms are recorded as follows: phanerophytes (P), chamaephytes (Ch), hemicryptophytes (H), geophytes (G), helophytes (He), hydrophytes (I).

### Native vs. non-native status

The assessment of native versus introduced status is based on the information provided by the literature sources and on-line data-bases (e.g. APD). A preliminary assessment for 131 taxa was published by Brundu and Camarda (2004). Species of uncertain status are considered cryptogenic (Carlton, 1996).

## Results and discussion

This checklist for Chad counts 2,460 entities (hereafter records or taxa), i.e. 2288 species (including 128 autonyms), 83 subspecies, 81 varieties, 8 forms. They belong to 151 Families, with 48.7% of the taxa belonging to the 6 largest families, i.e. 359 taxa in the *Poaceae* (14.6%), 335 in the *Fabaceae* (13.6%), 173 in the *Cyperaceae* (7.0%), 153 (6.2%) in the *Asteraceae*, 95 in the *Malvaceae* (3.9%) and 84 in the Rubiaceae (3.4%).

As stated in the introduction, the previous available information on the Flora of Chad reported a number of 1,600 entities (without a clear distinction between the number of species and subspecific entities). Actually the 4 main contributions for southern Chad account in the present checklist for 1,638 records, as follows: 1,520 reported by Lebrun et al. (1972), 57, 20, 41 reported, respectively by Lebrun and Gaston in their 3 additions to the original list (Lebrun and Gaston 1976, 1977, 1986). We include in the list also 121 records for the Tibesti from the work of Corti (1942), 374 recorded by Maire and Monod (1950), 136 by Scholz (1966), 539 records for the Ennedi from the work of Gillet (1968). The other records in the checklist derive therefore from GBIF information, from the other literature sources cited in the methods section, from the authors’ personal observations and from personal communications received for Zakouma National park from P. Poilecot.

A total number or 2,173 taxa (88.3%) are native to Chad, including 55 (2.2%) endemics, while 274 (11.0%) are alien to Chad, and 13 (0.5%) are considered cryptogenic, i.e. of uncertain status. This represents a considerable updated of the previous knowledge on the alien flora of Chad that counted for 131 taxa (5.3%) Brundu and Camarda (2004).

The majority of taxa in the checklist are perennials (1388, 56.4%), annuals account for 772 taxa (31.4%) and there are 300 taxa (12.2%) with other types of life histories (biannual, annual or biannual, annual or short-lived perennial and annual or perennial).

There are 657 therophytes (26.7%), 546 phanerophytes (22.2%), 378 hemicryptophytes (15.4%), 256 chamaephytes (10.4%), 160 geophytes (6.5%), 107 helophytes (4.3%), 104 hydrophytes (4.2%). A total of 252 taxa (10.2%) may have different life forms (e.g. terophytes or chamaephytes) and, in the past, were considered as *plurisaisonnières* by Gillet (1968). This author indicated 43 taxa of this type for the Ennedi.

Assessing biodiversity and understanding mechanisms of its changes are difficult in many areas of Africa because data are incomplete or lacking. Conserving biodiversity in Chad is challenging for several reasons including incomplete knowledge, differing and incomplete conceptual models of main ecological processes, major gaps in ecological and management knowledge, high variability in ecological responses to climate, altered vegetation regimes as a result of land-use history and climate change, and the increasing encroachment of open forest landscapes and several habitats by humans.

We hopefully expect that our checklist would refuel botanical researches in Chad and constitute a reliable basis for the necessary further studies and nature conservation planning.

## Appendix I

Checklist of accepted names. (doi: 10.3897/phytokeys.23.4752.app1) File format: Compressed Archive (.rar).

**Explanation note:** The checklist table is composed by 35 columns and 2460 rows (Microsoft Excel spreadsheet). A description of the content of each column is provided in an additional file (Microsoft Word document).

## Appendix II

Accepted names and synonyms. (doi: 10.3897/phytokeys.23.4752.app2) File format: Microsoft Excel documents (xls).

**Explanation note:** The synonym table is a complement to the checklist and it is composed by 7 columns and 16,750 rows (Microsoft Excel spreadsheet). A description of the content of each column is provided in an additional file (Microsoft Word document).
